# Burnout among primary healthcare workers during implementation of integrated mental healthcare in rural Ethiopia: a cohort study

**DOI:** 10.1186/s12960-019-0383-3

**Published:** 2019-07-18

**Authors:** Medhin Selamu, Charlotte Hanlon, Girmay Medhin, Graham Thornicroft, Abebaw Fekadu

**Affiliations:** 1 College of Health Sciences, School of Medicine, Department of Psychiatry, Addis Ababa University, PO Box 9086, Addis Ababa, Ethiopia; 2 Centre for Global Mental Health, Health Service and Population Research Department, Institute of Psychiatry, Psychology and Neuroscience, King’s College London, London, United Kingdom; 3 Centre for Innovative Drug Development and Therapeutic Trials for Africa (CDT-Africa), College of Health Sciences, Addis Ababa University, Addis Ababa, Ethiopia; 4 Aklilu Lemma Institute of Pathobiology, Addis Ababa University, Addis Ababa, Ethiopia; 5 Global Health & Infection Department, Brighton and Sussex Medical School, Brighton, United Kingdom; 6 Department of Psychological Medicine, Centre for Affective Disorders, Institute of Psychiatry, Psychology and Neuroscience, King’s College London, London, United Kingdom

**Keywords:** Job-related stress, Burnout, Professional satisfaction, Wellbeing, Healthcare workers, Primary healthcare, Ethiopia

## Abstract

**Background:**

The short-term course of burnout in healthcare workers in low- and middle-income countries has undergone limited evaluation. The aim of this study was to assess the short-term outcome of burnout symptoms in the context of implementation of a new mental health programme in a rural African district.

**Methods:**

We followed up 145 primary healthcare workers (HCWs) working in 66 rural primary healthcare (PHC) facilities in Southern Ethiopia, where a new integrated mental health service was being implemented. Burnout was assessed at baseline, i.e. when the new service was being introduced, and after 6 months. Data were collected through self-administered questionnaires, including the Maslach Burnout Inventory (MBI) and instruments measuring professional satisfaction and psychosocial factors. Generalised estimating equations (GEE) were used to assess the association between change in the core dimension of burnout (emotional exhaustion) and relevant work-related and psychosocial factors.

**Results:**

A total of 136 (93.8%) of HCWs completed and returned their questionnaires at 6 months. There was a non-significant reduction in the burnout level between the two time points. In GEE regression models, high depression symptom scores (adjusted mean difference (aMD) 0.56, 95% CI 0.29, 0.83, *p* < 0.01), experiencing two or more stressful life events (aMD 1.37, 95% CI 0.06, 2.14, *p* < 0.01), being a community health extension worker vs. facility-based HCW (aMD 5.80, 95% CI 3.21, 8.38, *p* < 0.01), perceived job insecurity (aMD 0.73, 95% CI 0.08, 1.38, *p* = 0.03) and older age (aMD 0.36, 95% CI 0.09, 0.63, *p* = 0.01) were significantly associated with higher levels of emotional exhaustion longitudinally.

**Conclusion:**

In the short-term, there was no significant change in the level of burnout in the context of adding mental healthcare to the workload of HCWs. However, longer term and larger scale studies are required to substantiate this. This evidence can serve as baseline information for an intervention development to enhance wellbeing and reduce burnout.

## Background

Mental healthcare is a neglected component of healthcare in most low- and middle-income countries (LMICs). As a consequence, the treatment gap even for serious mental disorders in LMICs is substantial [1]. The available mental health services are mostly centralised and led by a small number of specialists [2]. In recognition of the impossibility of reaching all in need through specialists, the World Health Organization (WHO) developed the Mental Health Gap Action Programme (mhGAP) [3]. This initiative provides interventions for improving access to care through task-shared care integrated into primary healthcare (PHC) [4]. There is some evidence indicating the feasibility [5], efficiency and cost-effectiveness [2] of this model of care provision, in addition to some evidence that PHC-based care may be less stigmatising [6]. Moreover, as PHC is the place where most people across the world receive healthcare [7], task-sharing is an attractive proposition for achieving Universal Health Coverage (UHC) [8, 9].

However, the acute shortage and maldistribution of HCWs in LMICs [10, 11] has major implications for the feasibility and acceptability of task-shared care. For example, in Ethiopia, the HCW to population ratio of 0.7 per 1000 population is much lower than the minimum that has been recommended by WHO (2.3 per 1000) [12]. This occurs in the context of a fragile healthcare system and a high disease burden, low healthcare resources and stressful work environment [13, 14]. Therefore, engaging HCWs in task-shared care for people with mental health problems may expose them to an additional burden with the potential for increased work-related stress and burnout. On the other hand, training HCWs in mental healthcare could reduce stress and burnout because they are better equipped to deal with a set of commonly encountered conditions and may also develop understanding of their own mental health needs.

Ethiopia has chosen to undertake the expansion of mental healthcare through integration into primary care as a major strategic agenda [15]. Although this will undoubtedly expand the responsibility of primary HCWs and may expose them to job-related health issues such as burnout, appropriate training support may increase their competence and confidence. The latter may enhance their wellbeing. Expanding the responsibility of HCWs may not increase the risk of burnout however significant the risk might be.

Burnout is a phenomenon resulting from exposure to continuous job-related stress [16]. It is understood to be a group of three sub-syndromes constituted as domains. The first domain is emotional exhaustion (EE), which is a feeling of being overextended and inability to be compassionate to others. The second domain is cynicism (CY) or depersonalisation in which the HCW distances him/herself from the patient and begins to see them as impersonal objects. The third domain is a reduced feeling of personal accomplishment (PA). EE is considered as the core element of burnout [17]. In this state, the provider evaluates his/her work negatively irrespective of patient outcomes [18, 19]. Burnout may have serious consequences for the quality of healthcare delivery, patient safety [20, 21] and the wellbeing of the HCW. For example, burnout has been associated with chronic fatigue [22], physical health problems such as low back pain [23], cardiovascular diseases [24], depression [25, 26], and substance abuse problems [27] in HCWs experiencing burnout.

Several factors have been found to be associated with the development of HCW burnout, including socio-demographic [28, 29], psychosocial [30], work-related factors, such as role ambiguity and lack of experience [31], and team relationships [32]. Lower job satisfaction and employee’s negative attitude towards their job are also linked with burnout [33]. Several studies have indicated that African HCWs have low job satisfaction [34–36], which may increase the risk of burnout [37]. Development of burnout can have an impact on the HCW’s motivation [38], intention to stay in the position [39], compassion to [40], and communication with, patients [41] and service quality [42, 43].

However, HCW wellbeing in general, and level of burnout and professional satisfaction in the context of mental health service integration in particular, is a little-investigated area in LMICs especially in sub-Saharan Africa [23, 41, 44]. In addition, most of the available studies on the subject in LMIC have used a cross-sectional study design [23, 31, 32, 45, 46].

In our previous studies, we have explored the conceptualisaton of burnout, job-related wellbeing and wellbeing [47] and cross-sectionally estimated the magnitude of burnout [48] in HCWs in a rural Ethiopian district. In this study, we aim to evaluate change in burnout symptoms and associated factors among rural primary HCWs during the implementation of a new integrated mental health service.

## Method

### Study design

A cohort study was conducted with HCWs working in governmental health facilities in a rural Ethiopia district and followed up for 6 months.

### Setting

The study was conducted over 6 months, between July 2014 and December 2014, in the Sodo district of the Gurage zone, Ethiopia. Details of the setting have been reported previously and will be described in brief [48]. During the study, the district had 54 rural and four urban sub districts and an estimated population of 161 952 (79 356 men; 82 596 women) [49].

Sodo district was chosen for this study because the district hosts the PRogramme for Improving of Mental health care (PRIME) where the integration of mental health services in PHCs through task-sharing was taking place [50]. Primary care in the integration site consisted of 66 facilities that include 8 primary health centres and 58 health posts. The health centres are staffed by health officers (practitioners with 3 to 4 years of clinical training), nurses and midwifes. The health posts are led by community-based health extension workers (HEWs). HEWs are high school graduates with 1-year training. Their work focuses on prevention and health promotion, and they mainly provide a house to house service. The Ethiopian healthcare system has three tiers. The first level is made of rural health centres and primary hospitals, the second level is general hospitals and the third one is specialised referral hospitals. The workforces in the first level consists of health officers, nurses and midwives with the expectation of serving 15 000–25 000 community members in rural area and up to 40 000 people in urban areas [51]. Mental health service training guided by mhGAP intervention guide manual [52] was provided to all facility- and community-based HCWs based on an initial mental healthcare plan [53].

### Study participants

**Fig. 1:**
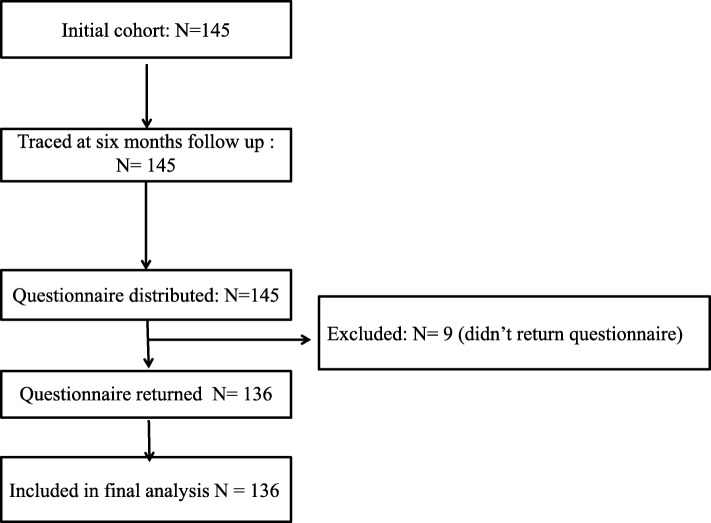
Cohort flow diagram

To be included, participants had to fulfil the following criteria: (1) work in a PHC setting in the Sodo district, (2) have direct engagement in health service provision, (3) be already trained in mhGAP, and (4) have a minimum of 3-month work experience in the same workplace or a similar setting. The details of the recruitment and 6 months follow-up are presented in Fig. 1.

Participants were grouped based on their primary place of work as facility-based or community-based HCWs. Facility-based HCWs are health officers, nurses and midwives, and the community-based HCWs.

### Outcome variables and measures

The outcome measure was used at baseline and 6 months follow-up. The primary outcome was the change in level of burnout, and the secondary outcome was the change in the professional satisfaction. The Maslach Burnout Inventory (MBI) human service survey version, a widely used burnout measure, was the main scale used as a measure of burnout [54]. The scale was 22 items [55] focused at assessing emotional exhaustion (EE), cynicism (CY) and reduced personal accomplishment (PA) domains. The interpretation of the MBI score relies on the total score of each domain. High score of EE and CY domains and low score of PA domain represent burnout [56]. EE score of 0–16 and CY score of 0–6 are considered low or normal scores, while scores above 16 for EE and scores above 6 for CY are considered moderate or high score. [57]. The psychometric property of the MBI was generally acceptable and comparable with other studies [17]. In our baseline cross-sectional study, we assessed the psychometric properties of MBI. The overall internal consistency of the 22 items was adequate (Cronbach’s *α* = 0.70). The domain specific internal consistency was acceptable for EE (Cronbach’s *α* = 0.75) and PA domains (Cronbach’s *α* = 0.80). However, the internal consistency of the domain CY was unacceptable (Cronbach’s *α* = 0.18). We have, therefore, excluded the CY domain from all the analysis.

Professional satisfaction was also assessed as a secondary outcome using the Job Satisfaction Questionnaire (JSQ). This is a 15-item scale [58] rated on a 7-point scale with responses ranging from extremely dissatisfied (coded as 1) to extremely satisfied (coded as 7). The JSQ evaluates intrinsic and extrinsic job satisfaction and can also be summarised as an overall job satisfaction score [58]. It has good overall internal consistency (Cronbach’s *α* = 0.89).

### Exposure variables

Socio-demographic and work-related characteristics were assessed using a structured questionnaire locally designed to collect information on age, gender, marital status, educational level, place of work, type of work and work experience.

Psychosocial factors: Depression was assessed using the locally validated nine-item Patient Health Questionnare (PHQ-9) [59–62]; a cutoff point 5 and above was used to define probable depression. Social support was assessed using the three-item Oslo Social Support Scale (OSS) [63], and the list of threatening experiences (LTE) was used to identify the experience of stressful life events in the previous 6 months [64, 65]. The Alcohol Use Disorders Identification Test (AUDIT) was used to evaluate the alcohol consumption and drinking behaviour of the participants [66].

The Job Content Questionnaire (JCQ) was used to measure work-related factors; it is a 27-item scale with three main domains that focus on the individual’s decision latitude, psychological demand and social support. This scale also assesses the physical demands of the job and job insecurity [62]. The decision latitude consists of two things; the first is job skill discretion, that is, the opportunity to use a given skill in executing the job. The second is decision-making authority which is the degree of making autonomous decision regarding how to do the job.

### Procedures for data collection

At baseline, the instruments were pilot tested in a neighbouring area of Silte zone on 25 primary HCWs (the data have not been included in this report). All the questionnaires were in self-report format. At both time points (i.e. at baseline or T1 and 6 months follow-up or T2), MS provided information about the study and how to fill the self-reported questionnaires and distributed the questionnaires to the participants. Participants returned the completed questionnaires in a sealed envelope.

### Data management and analysis

Epi Data version 3.1 (http://www.epidata.dk/) was used for double data entry, and Stata version 13 (StataCorp, 1985–2013) was used for statistical analysis. In addition to simple descriptive statistics, longitudinally collected data were analysed using generalised estimating equation (GEE). Change in EE was considered as the main outcome representing burnout and professional satisfaction as secondary outcome, whereas demographic, psychosocial and work-related factors were treated as covariates. Factors associated with change in the major outcomes variables were explored. In the descriptive section, burnout result was summarised by adding those who scored moderate to high EE and low PA. Those endorsing either one of the two or both domains of burnout were grouped as having burnout.

### Ethical approval

This study was approved by the Addis Ababa University, College of Health Sciences Institutional Review Board (protocol number 011/14/Psy). Written informed consent was obtained from all study participants after providing information about the aim of the study to the participants. Hard copies of the data were kept in a locked cabinet, and soft copies were kept in password-protected computers to maintain anonymity of participants and confidentiality of information. Care was available after the interviews for those experiencing distress or any sort of emotional problem, including suicidal ideation.

## Results

### Socio-demographic characteristic of the study participants

From the 145 HCWs recruited at baseline, 136 (93.8%) competed the 6 months follow-up assessment. At baseline, most participants were female (61.0%, *n* = 80), under 35 years of age (93.0% *n* = 110), single (63.0%, *n* = 83) and nurses (51.0%, *n* = 70). About a third of the participants were HEWs (33.0%, *n* = 45) working in the community while two thirds were facility-based (Table 1). Loss to follow-up was not significantly associated with the outcome of interest or demographic factors.

**Table 1:** Baseline demographic characteristics of study participants

	Characteristics	Number	Percent
Gender (*n* = 148)	Male	57	39
	Female	91	61
Age (*n* = 145)	< 25	57	39
	25–35	79	55
	> 35	9	6
Marital status (*n* = 145)	Single	96	66
	Married	49	34
Professional group (*n* = 148)	Nurse	75	51
	Health officer	9	6
	Midwife	13	9
	Health extension worker	51	34
Place of work (*n* = 145)	Urban	64	44
	Rural	81	56

### Overall burnout score

The proportion categorised as having moderate to high burnout in both domains, EE and reduced PA, was relatively low at both time points: 3.8% (*n* = 5) at baseline and 4.6% (*n* = 6) at the 6 months follow-up time point (*p* value = 0.765) (Table 2). There was statistically non-significant change in the proportion endorsing either of the sub domains (42.9%, *n* = 57 versus 46.6%, *n* = 61, *p* value = 0.584).

**Table 2:** Burnout domain score status of study participants stratified by the study period

Emotional exhaustion (EE) and reduced personal accomplishment (PA) scores	Baseline		Endline	
	*N*	%	*N*	%
Low score in both domains (i.e. EE and PA)	71	53.4	64	48.8
Moderate to high score only in one domain (i.e. in EE or in PA but not both)	57	42.9	61	46.6
Moderate to high score in the two domains (i.e. EE and PA)	5	3.8	6	4.6

### Domain-specific burnout scores

#### Emotional exhaustion (EE)

The median (interquartile range) scores were 3 (0, 23) at baseline and 2 (0, 29) at follow-up (Table 4). The proportion of participants with elevated EE score was almost identical at the two time points (T1 = 7.7% (*n* = 11) and T2 = 7.5% (*n* = 10)), with no statistically significant difference. At both time points, community-based HEWs experienced higher EE than facility-based HCWs (*p* = 0.12). However, there was no statistically significant change in EE scores over time by professional group (Table 3). At both time points, women were more likely to report moderate to high EE: 11.2% (*n* = 10) in women vs. 1.9% (*n* = 1) in men at T1 and 10.1% (*n* = 8) in women at T1 vs. 3.9% (*n* = 2) in men at T2.

**Table 3:** Baseline and endline burnout scores of study participants using emotional exhaustion and personal accomplishment domains

		Baseline			Endline		
Burnout domains	Score	Overall *N* (%)	Community-based HCWs *N* (%)	Facility-based HCWs *N* (%)	Overall *N* (%)	Community-based HCWs *N* (%)	Facility-based HCWs *N* (%)
Emotional exhaustion	Moderate to high burnout*	11 (7.7)	9 (18.0)	2 (2.2)	10 (7.5)	5 (11.4)	5 (5.6)
	Low burnout	131 (92.2)	41 (82.0)	90 (97.8)	123 (92.5)	39 (88.6)	84 (94.4)
Personal accomplishment	Moderate to high burnout	59 (43.7)	22 (50.0)	37 (40.6)	64 (48.5)	21 (50.0)	43 (47.8)
	Low burnout	76 (56.3)	22 (50.0)	54 (59.3)	68 (61.9)	21 (50.0)	47 (52.2)

*HCWs* healthcare workers

*Significant difference between community- and facility-based HCWs at baseline

#### Personal accomplishment (PA)

The median (interquartile range) scores were 34 (0, 47) at baseline and 32 (0, 43) at 6 months follow-up (Table 4). At T1, 43.7% (*n* = 59) and, at T2, 48.5% (*n* = 64) participants reported reduced feeling of PA defined by the standard authors’ cut-off. The baseline differences in PA were maintained at 6 months follow-up in the various professional groups without a statistically significant change over time.

**Table 4:** Median and 25th and 75th percentile ranges of burnout, job content and satisfaction of study participants stratified by the time of survey

Burnout and job-related factors	Baseline		Endline	
Burnout domain	Median	IQR (25th, 75th)	Median	IQR (25th, 75th)
Emotional exhaustion	3	(0, 23)	2	(0, 29)
Personal accomplishment	34	(0, 47)	32	(0, 43)
Job content domain				
Job skill discretion	34	(26, 44)	34	(32, 36)
Job decision-making authority	36	(24, 48)	36	(20, 48)
Job demands	34	(24, 46)	34	(26, 42)
Job decision latitude	72	(54, 90)	70	(44, 90)
Co-worker support	12	(8, 16)	12	(7, 16)
Supervisor support	12	(5, 17)	12	(5, 17)
Job insecurity	5	(3, 9)	5	(3, 8)
Job satisfaction				
Intrinsic job satisfaction	35	(15, 48)	35	(17, 46)
Extrinsic job satisfaction	35	(15, 54)	35	(18, 53)
Overall job satisfaction	72	(32, 99)	70	(38, 98)

### Secondary outcomes

#### Job satisfaction

There was no significant change in intrinsic and extrinsic job satisfaction scores, and consequently of overall job satisfaction, between T1 and T2 (Table 4).

### Explanatory variables

#### Job content

There was no marked change in the job content measure score in any of the domains of job content and control between T1 and T2 (Table 4).

#### Depressive symptoms

*The* proportion of HCWs with a high score on the PHQ has reduced from 20.6% (*n* = 29) at T1 to 11.2% (*n* = 5) at T2, which was statistically significant *p* = 0.03.

### Generalised estimating equation (GEE) model

In the crude model, a longitudinal higher average EE score was associated with female gender, lower co-workers support, lower supervisor support, low job skill discretion (which is the opportunity to use one’s specific skill), and lower intrinsic and extrinsic job satisfaction. High PHQ score or elevated depressive symptoms (adjusted mean difference (aMD) = 0.56, 95% CI 0.29, 0.83, *p* < 0.01), having experience of a stressful life event (aMD = 1.37, 95% CI 0.60, 2.14, *p* < 0.01), being community-based HCWs (aMD = 5.80, 95% CI 3.21, 8.38, *p* < 0.01) and high job insecurity (aMD = 0.73 95% CI 0.08, 1.38, *p* = 0.03) were significantly associated with higher EE scores in both crude and fully adjusted models. Increase in age was significantly associated with higher EE in the fully adjusted model (aMD = 0.36, 95% CI 0.09, 0.63, *p* = 0.01 (Table 5).

**Table 5:** Association of demographic, psychosocial and work-related factors with emotional exhaustion using generalised estimating equations

		Crude model			Fully adjusted model*		
Selected characteristics	Response categories	Mean difference	95% CI	*p* value	Mean difference	95% CI	*p* value
Time		− 0.04	− 0.27, 0.17	0.68	0.16	− 0.09, 0.42	0.20
Sex	Male	Ref					
	Female	2.68	0.95, 4.41	< 0.01	− 1.28	− 4.04, 1.48	0.36
Age		− 0.003	− 0.18, 0.17	0.97	0.40	0.12, 0.67	< 0.01
Marital status	Single	Ref					
	Married	0.77	− 1.02, 2.56	0.40	− 1.07	− 3.34, 1.19	0.35
Place of work	Urban	Ref					
	Rural	1.51	− 0.18, 3.20	0.08	1.63	− 0.73, 4.01	0.17
Psychosocial factors	PHQ score*	0.70	0.53, 0.87	< 0.01	0.58	0.31, 0.84	< 0.01
	Social support*	0.32	− 0.19, 0.85	0.22	− 0.02	− 0.63, 0.59	0.95
	Stressful life events*	1.43	0.83, 2.02	< 0.01	1.37	0.65, 2.09	< 0.01
Profession	Facility-based healthcare workers	Ref					
	Community-based healthcare workers	5.71	4.0, 7.39	< 0.01	5.65	2.85, 8.44	< 0.01
Year of service		− 0.23	− 0.52, 0.05	0.11	− 0.18	− 0.55, 0.18	0.32
Job satisfaction	Intrinsic job satisfaction*	− 0.32	− 0.43, − 0.21	< 0.01	− 0.02	− 0.23, 0.20	0.84
	Extrinsic job satisfaction*	− 0.22	− 0.31, − 0.14	< 0.01	− 0.14	− 0.36, 0.06	0.16
Job content and control	Job skill discretion*	− 0.25	− 0.46, − 0.03	0.02	− 0.22	− 0.51,0.05	0.12
	Job decision-making attribute*	− 0.06	− 0.19, 0.08	0.42	0.01	− 0.18, 0.19	0.97
	Job demand*	− 0.02	− 0.19, 0.16	0.84	0.06	− 0.15, 0.27	0.57
	Co-workers support*	− 0.57	− 1.05. -0.09	0.02	− 0.50	− 1.29, 0.29	0.21
	Supervisors support*	− 0.44	− 0.75, − 0.14	< 0.01	0.22	− 0.21, 0.67	0.32

*Mean difference of the EE score associated with continuous predictor variables indicate (a) 1-year increase in age (b), 1-point increase in PHQ-9 total score, (c) 1-point increase in social support score, (d) 1-point increase in total score of stressful life events, (e) a 1-year increase in the service years, (f) 1-point increase in the intrinsic job satisfaction score, (g) 1-point increase in the extrinsic job satisfaction score, (h) a 1-point increase in the “job skill discretion score”, (i) a 1-point increase in the “job decision-making attribute score”, (j) a 1-point increase in the “job demand score”, (k) a 1-point increase in the “co-workers support score”, (l) a 1-point increase in the “supervision support score”

## Discussion

In this short-term cohort study of primary HCWs in Ethiopia, carried out during implementation of a new integrated mental healthcare service, we found no significant change in burnout or professional satisfaction. Community-based HCWs, females, older staff, those with higher depressive symptom scores, those having two or more threatening life experiences at baseline and those with high perceived job insecurity were at increased risk of emotional exhaustion. Since health service in general and mental health service in particular is dependent on the HCWs wellbeing [67], the results of this study may inform intervention development to promote HCWs’ wellbeing and facilitate the integration of mental health service in other PHCs.

As far as we know, this is the first study of its kind conducted in the context of implementation of integrated mental healthcare and one of the few longitudinal studies on HCW burnout in a LMIC. The results indicate that burnout level in HCWs did not increase or worsen during early integration stages of a new mental health programme. The steady nature of burnout was also reported in the findings of a longitudinal study on burnout of teachers [68]. In other contexts, for example, in humanitarian work context, burnout and anxiety levels may rise in the initial stage and then settle at a steady level over 3 to 6 months of follow-up [69].

Although the work itself might be additional burden, the enhancement of knowledge through the mhGAP for the diagnosis and treatment of mental disorders [4] may have boosted their confidence in dealing with those with complex health needs and thus counter balanced potential negative impact of additional work load. This can help them to have more confidence and skill in their work, especially while handling people in need of mental health services.

There might be some overlap between the constructs of depression and EE; some studies have reported depression as a predictor of burnout [25] while other studies fail to find association between the two [70]. Our study found association between EE and depression, but only depression reduced significantly during the follow-up suggesting perhaps the two constructs might be different.

Although some studies have found association between social support and burnout [71], we did not find such association. However, being a community-based health worker (HEW) was significantly associated with burnout. This may have arisen from the difference in the job demand as well as control over one’s job [72]. Lower level of education might also have contributed as suggested in an Iranian health workers study [29].

In contrast to many African studies [34–36], including Ethiopian studies [73, 74], in this study, we found that there is a good level of professional satisfaction among the HCWs which was steady over the 6-month follow-up period. This is a promising and favourable situation for scaling up of not only mental healthcare but also healthcare in general as envisaged in the sustainable development goals [74].

The study has important limitations that impact interpretation. First, the sample size was relatively small although all health workers from the district were included. Secondly, there is a potential for social desirability bias due to stigma against reporting burnout and associated concepts. Thirdly, although some pre-testing and reliability checks were carried out, the outcome measures had not been validated formally in the study setting. Finally, linked to the above problem is the fact that one of the three domains (CY) was excluded from analysis because of the low internal consistency.

## Conclusion

Providing mental health services did not appear to have a significant short-term negative effect on the level of burnout and professional satisfaction of primary HCWs.

The results of this study can be used as baseline information to develop an intervention aimed at improving primary HCWs’ wellbeing in Ethiopia. It can also be used as a signal of the existence of burnout for policy makers and healthcare managers. Further studies are needed to assess HCWs’ wellbeing in a holistic manner and its effects on the health service, patient satisfaction and outcome. Intervention to enhance wellbeing should give priority to community healthcare workers given the higher level of burnout and nature of their job.

## Data Availability

The data is part of multi country study, Programme for Improving Mental Healthcare (PRIME). Because of the ongoing nature of the study, we are not currently able to make the data publically available. However, data will be made available once the study is completed next year.

## Abbreviations

aMD: Adjusted mean difference
AUDIT: Alcohol Use Disorders Identification Test
CY: Cynicism
EE: Emotional exhaustion
GEE: Generalised estimating equations
HCW: Healthcare worker
HEW: Health extension worker
HSTP: Health Sector Transformation Plan
JCQ: Job Content Questionnaire
JSQ: Job Satisfaction Questionnaire
LMIC: Low- and middle-income countries
LTE: List of threatening experiences
MBI: Maslach Burnout Inventory
mhGAP: Mental Health Gap Action Programme
OSS: Oslo Social Support scale
PA: Personal accomplishment
PHC: Primary health care
PHQ: Patient Health Questionnaire
PRIME: Programme for Improving Mental Healthcare
SDG: Sustainable development goals
SNNPR: Southern Nations, Nationalities and Peoples Regions
T1: Baseline
T2: Six months follow-up
UHC: Universal Health Coverage
WHO: World Health Organization

## References

[1] Demyttenaere K, Bruffaerts R, Posada-Villa J, Gasquet I, Kovess V, Lepine J, Angermeyer MC, Bernert S, Morosini P, Polidori G (2004). Prevalence, severity, and unmet need for treatment of mental disorders in the World Health Organization World Mental Health Surveys. Jama.

[2] Joshi R, Alim M, Kengne AP, Jan S, Maulik PK, Peiris D, Patel AA (2014). Task shifting for non-communicable disease management in low and middle income countries–a systematic review. PLoS One.

[3] Organization WH. Mental health global action programme (mhGAP): close the gap, dare to care. Geneva: World Health Organization; 2002.

[4] Keynejad Roxanne C, Dua Tarun, Barbui Corrado, Thornicroft Graham (2017). WHO Mental Health Gap Action Programme (mhGAP) Intervention Guide: a systematic review of evidence from low and middle-income countries. Evidence Based Mental Health.

[5] Organization WH. The World Health Report 2001: Mental health: new understanding, new hope: World Health Organization; 2001.

[6] Organization WH (2008). Integrating mental health into primary care: a global perspective.

[7] Starfield B, Shi L, Macinko J (2005). Contribution of primary care to health systems and health. Milbank Q.

[8] Frenk J (2015). Leading the way towards universal health coverage: a call to action. Lancet.

[9] Frenk J (2009). Reinventing primary health care: the need for systems integration. Lancet.

[10] Aluttis C, Bishaw T, Frank MW (2014). The workforce for health in a globalized context–global shortages and international migration. Glob Health Action.

[11] Dussault G, Franceschini MC (2006). Not enough there, too many here: understanding geographical imbalances in the distribution of the health workforce. Hum Resour Health.

[12] Awases M, Nyoni J, Bessaoud K, Diarra-Nama AJ, Ngenda CM, Awases M. Development of Human Resources for Health in the WHO African Region: current situation and way forward. African Heal Monit 2010, 12:22–29. Accessed 1 Nov 2018.

[13] Mills A (2014). Health care systems in low-and middle-income countries. N Engl J Med.

[14] Organization WH (2006). Working together for health: the World health report 2006: policy briefs.

[15] Health FDRoEMo (2012). National Mental Health Strategy 2012/13–2015/16. Health Mo ed.

[16] Freudenberger HJ (1974). Staff burn-out. J Soc Issues.

[17] Worley JA, Vassar M, Wheeler DL, Barnes LL (2008). Factor structure of scores from the Maslach Burnout Inventory: a review and meta-analysis of 45 exploratory and confirmatory factor-analytic studies. Educ Psychol Meas.

[18] Maslach C (1979). Burned-out. Can J Psychiatr Nurs.

[19] Maslach C, Schaufeli WB, Leiter MP (2001). Job burnout. Annu Rev Psychol.

[20] Dewa CS, Loong D, Bonato S, Trojanowski L, Rea M (2017). The relationship between resident burnout and safety-related and acceptability-related quality of healthcare: a systematic literature review. BMC Med Educ.

[21] Dyrbye LN, Shanafelt TD, Sinsky CA, Cipriano PF, Bhatt J, Ommaya A, West CP, Meyers D (2017). Burnout among health care professionals: a call to explore and address this underrecognized threat to safe, high-quality care. NAM (National Academy of Medicine) Perspective.

[22] Rose D, Seidler A, Nübling M, Latza U, Brähler E, Klein E, Wiltink J, Michal M, Nickels S, Wild P (2017). Associations of fatigue to work-related stress, mental and physical health in an employed community sample. BMC Psychiatry.

[23] Boran A, Shawaheen M, Khader Y, Amarin Z, Hill Rice V (2011). Work-related stress among health professionals in northern Jordan. Occup Med.

[24] Melamed S, Kushnir T, Shirom A (1992). Burnout and risk factors for cardiovascular diseases. Behav Med.

[25] Bianchi R, Schonfeld IS, Laurent E (2015). Is burnout separable from depression in cluster analysis? A longitudinal study. Soc Psychiatry Psychiatr Epidemiol.

[26] Fong TC, Ho RT, Au-Yeung FS, Sing C, Law K, Lee L, Ng S (2016). The relationships of change in work climate with changes in burnout and depression: a 2-year longitudinal study of Chinese mental health care workers. Psychol Health Med.

[27] Jackson ER, Shanafelt TD, Hasan O, Satele DV, Dyrbye LN (2016). Burnout and alcohol abuse/dependence among U.S. medical students. Acad Med.

[28] Abdulla L, Al-Qahtani D, Al-Kuwari M (2011). Prevalence and determinants of burnout syndrome among primary healthcare physicians in Qatar. S Afr Fam Pract.

[29] Kabir MJ, Heidari A, Etemad K, Gashti AB, Jafari N, Honarvar MR, Ariaee M, Lotfi M (2016). Job burnout, job satisfaction, and related factors among health care workers in Golestan Province, Iran. Electron Physician.

[30] Bakker AB, Schaufeli WB, Demerouti E, Janssen PP, Van Der Hulst R, Brouwer J (2000). Using equity theory to examine the difference between burnout and depression.

[31] Aloulou J, Damak R, Masmoudi F, Sidhom O, Amami O (2013). Burn out in health care providers: a Tunisian study about 142 nurses. Tunis Med.

[32] Lasebikan VO, Oyetunde MO (2012). Burnout among nurses in a Nigerian general hospital: prevalence and associated factors. ISRN Nurs.

[33] Dall'Ora C, Griffiths P, Ball J, Simon M, Aiken LH (2015). Association of 12 h shifts and nurses’ job satisfaction, burnout and intention to leave: findings from a cross-sectional study of 12 European countries. BMJ Open.

[34] van der Doef M, Mbazzi FB, Verhoeven C (2012). Job conditions, job satisfaction, somatic complaints and burnout among East African nurses. J Clin Nurs.

[35] Delobelle P, Rawlinson JL, Ntuli S, Malatsi I, Decock R, Depoorter AM (2011). Job satisfaction and turnover intent of primary healthcare nurses in rural South Africa: a questionnaire survey. J Adv Nurs.

[36] Blaauw D, Ditlopo P, Maseko F, Chirwa M, Mwisongo A, Bidwell P, Thomas S, Normand C (2013). Comparing the job satisfaction and intention to leave of different categories of health workers in Tanzania, Malawi, and South Africa. Glob Health Action.

[37] Dugani S, Afari H, Hirschhorn LR, Ratcliffe H, Veillard J, Martin G, Lagomarsino G, Basu L, Bitton A (2018). Prevalence and factors associated with burnout among frontline primary health care providers in low-and middle-income countries: a systematic review. Gates Open Res.

[38] Engelbrecht S. Motivation and burnout in human service work: the case of midwifery in Denmark: Roskilde University, Faculty of Psychology, Philosophy and Science Studies; 2005.

[39] Arslan Yurumezoglu H, Kocaman G (2016). Predictors of nurses’ intentions to leave the organisation and the profession in Turkey. J Nurs Manag.

[40] Mitchel Jennifer A., Antoniak Silvio, Lee Joo-Hyeon, Kim Sae-Hoon, McGill Maureen, Kasahara David I., Randell Scott H., Israel Elliot, Shore Stephanie A., Mackman Nigel, Park Jin-Ah (2016). IL-13 Augments Compressive Stress–Induced Tissue Factor Expression in Human Airway Epithelial Cells. American Journal of Respiratory Cell and Molecular Biology.

[41] Ratanawongsa N, Roter D, Beach MC, Laird SL, Larson SM, Carson KA, Cooper LA (2008). Physician burnout and patient-physician communication during primary care encounters. J Gen Intern Med.

[42] Chao M, Shih CT, Hsu SF (2016). Nurse occupational burnout and patient-rated quality of care: the boundary conditions of emotional intelligence and demographic profiles. Jpn J Nurs Sci.

[43] Nantsupawat A, Nantsupawat R, Kunaviktikul W, Turale S, Poghosyan L (2016). Nurse burnout, nurse-reported quality of care, and patient outcomes in Thai hospitals. J Nurs Scholarsh.

[44] Karsavuran Seda, Kaya Sıdıka (2015). The relationship between burnout and mobbing among hospital managers. Nursing Ethics.

[45] Ashtari Z, Farhady Y, Khodaee M (2009). Relationship between job burnout and work performance among Iranian mental health professionals. Afr J Psychiatry.

[46] Kazmi R, Amjad S, Khan D (2008). Occupational stress and its effect on job performance a case study of medical house officers of district Abbottabad. J Ayub Med Coll Abbottabad.

[47] Selamu M, Thornicroft G, Fekadu A, Hanlon C (2017). Conceptualisation of job-related wellbeing, stress and burnout among healthcare workers in rural Ethiopia: a qualitative study. BMC Health Serv Res.

[48] Selamu M, Hanlon C, Medhin G, Thornicroft G, Fekadu A. Burnout and professional satisfaction of primary healthcare workers in rural Ethiopia (In press). BMC Public Health. 2018.

[49] Fekadu A, Medhin G, Selamu M, Hailemariam M, Alem A, Giorgis T, Breuer E, Lund C, Prince M, Hanlon C (2014). Population level mental distress in rural Ethiopia. BMC Psychiatry.

[50] Lund C, Tomlinson M, De Silva M, Fekadu A, Shidhaye R, Jordans M, Petersen I, Bhana A, Kigozi F, Prince M (2012). PRIME: a programme to reduce the treatment gap for mental disorders in five low- and middle-income countries. PLoS Med.

[51] FMOH. National Health Sector Transformation Plan. Health Mo ed. Addis Ababa: Fedral ministry of health; 2015.

[52] Organization WH. mhGAP intervention guide for mental, neurological and substance use disorders in non-specialized health settings: mental health Gap Action Programme (mhGAP)–version 2.0. World Health Organization; 2016. 27786430

[53] Fekadu A, Hanlon C, Medhin G, Alem A, Selamu M, Giorgis TW, Shibre T, Teferra S, Tegegn T, Breuer E (2016). Development of a scalable mental healthcare plan for a rural district in Ethiopia. Br J Psychiatry.

[54] SCHAUFELI W, GREENGLASS E (2001). Introduction to special issue on burnout and health. Psychol Health.

[55] Kalliath T, O’Driscoll M, Gillespie D, Bluedorn A (2000). A test of the Maslach burnout inventory in three samples of healthcare professionals. Work Stress.

[56] Schaufeli WB, Enzmann D, Girault N (1993). Measurement of burnout: a review. Professional burnout: recent developments in theory and research.

[57] Maslach C, Jackson S, Leiter MP (1996). Maslach burnout inventory manual.

[58] Stride C, Wall TD, Catley N. Measures of job satisfaction, organisational commitment, mental health and job related well-being: a benchmarking manual: Wiley; 2008.

[59] Hanlon C, Medhin G, Selamu M, Breuer E, Worku B, Hailemariam M, Lund C, Prince M, Fekadu A (2015). Validity of brief screening questionnaires to detect depression in primary care in Ethiopia. J Affect Disord.

[60] Gelaye B, Williams MA, Lemma S, Deyessa N, Bahretibeb Y, Shibre T, Wondimagegn D, Lemenhe A, Fann JR, Vander Stoep A (2013). Validity of the patient health questionnaire-9 for depression screening and diagnosis in East Africa. Psychiatry Res.

[61] Kroenke K, Spitzer RL, Williams JB, Löwe B (2010). The patient health questionnaire somatic, anxiety, and depressive symptom scales: a systematic review. Gen Hosp Psychiatry.

[62] Leka S, Jain A, Organization WH (2010). Health impact of psychosocial hazards at work: an overview.

[63] Kroll L, Lampert T (2011). Unemployment, social support and health problems. Dtsch Arztebl Int Med.

[64] Motrico E, Moreno-Kustner B, de Dios Luna J, Torres-Gonzalez F, King M, Nazareth I, Monton-Franco C, Gilde Gomez-Barragan MJ, Sanchez-Celaya M, Diaz-Barreiros MA (2013). Psychometric properties of the List of Threatening Experiences--LTE and its association with psychosocial factors and mental disorders according to different scoring methods. J Affect Disord.

[65] Brugha T, Bebbington P, Tennant C, Hurry J (1985). The List of Threatening Experiences: a subset of 12 life event categories with considerable long-term contextual threat. Psychol Med.

[66] Babor TF, Higgins-Biddle JC, Saunders JB, Monteiro MG. The alcohol use disorders identification test. Guidelines for use in primary health care Geneva: World Health Organization; 1992.

[67] Petersen I (2000). Comprehensive integrated primary mental health care for South Africa. Pipedream or possibility. Soc Sci Med.

[68] Hultell D, Bo Melin J, Gustavsson P (2013). Getting personal with teacher burnout: a longitudinal study on the development of burnout using a person-based approach. Teach Teach Educ.

[69] Cardozo BL, Crawford CG, Eriksson C, Zhu J, Sabin M, Ager A, Foy D, Snider L, Scholte W, Kaiser R (2012). Psychological distress, depression, anxiety, and burnout among international humanitarian aid workers: a longitudinal study. PLoS One.

[70] Bianchi R, Schonfeld IS, Schonfled S, Laurent E (2015). Burnout does not help predict depression among French school teachers. Scand J Work Environ Health.

[71] Hamaideh SH (2011). Burnout, social support, and job satisfaction among Jordanian mental health nurses. Issues Ment Health Nurs.

[72] Rodríguez I, Bravo MJ, Peiró JM, Schaufeli W (2001). The demands-control-support model, locus of control and job dissatisfaction: a longitudinal study. Work Stress.

[73] Semachew A, Belachew T, Tesfaye T, Adinew YM (2017). Predictors of job satisfaction among nurses working in Ethiopian public hospitals, 2014: institution-based cross-sectional study. Hum Resour Health.

[74] Manyazewal T, Matlakala MC (2017). Beyond patient care: the impact of healthcare reform on job satisfaction in the Ethiopian public healthcare sector. Hum Resour Health.

